# Stage 1 Registered Report: Effect of deficient phagocytosis on neuronal survival and neurological outcome after temporary middle cerebral artery occlusion (tMCAo)

**DOI:** 10.12688/f1000research.12537.3

**Published:** 2018-05-16

**Authors:** Julius V. Emmrich, Jonas J. Neher, Philipp Boehm-Sturm, Matthias Endres, Ulrich Dirnagl, Christoph Harms

**Affiliations:** 1 Department of Experimental Neurology and Center for Stroke Research, Charité – Universitätsmedizin Berlin, Corporate Member of Freie Universität Berlin, Humboldt-Universität zu Berlin, and Berlin Institute of Health, Berlin, Germany; 2 Department of Cellular Neurology, Hertie Institute for Clinical Brain Research, University of Tübingen, Tübingen, Germany; 3 German Center for Neurodegenerative Diseases (DZNE), Tübingen, Germany; 4 NeuroCure Cluster of Excellence and Charité Core Facility 7T Experimental MRIs, Charité – Universitätsmedizin Berlin, Berlin, Germany; 5 German Center for Neurodegenerative Diseases (DZNE), Berlin, Germany; 6 QUEST – Center for Transforming Biomedical Research, Berlin Institute of Health (BIH), Berlin, Germany

**Keywords:** stroke, neuroinflammation, phagocytosis, phagoptosis, middle cerebral artery occlusion, MCAo, microglia

## Abstract

Stroke is a major cause of death and disability worldwide. In addition to neuronal death resulting directly from energy depletion due to lack of blood supply, inflammation and microglial activation following ischemic brain injury has been increasingly recognized to be a key contributor to the pathophysiology of cerebrovascular disease. However, our understanding of the cross talk between the ischemic brain and the immune system is limited. Recently, we demonstrated that following focal ischemia, death of mature viable neurons can be executed through phagocytosis by microglial cells or recruited macrophages, i.e. through phagoptosis. It was shown that inhibition of phagocytic signaling pathways following endothelin-1 induced focal cerebral ischemia leads to increased neuronal survival and neurological recovery. This suggests that inhibition of specific phagocytic pathways may prevent neuronal death during cerebral ischemia. To further explore this potential therapeutic target, we propose to assess the role of phagocytosis in an established model of temporary (45min) middle cerebral artery occlusion (tMCAo), and to evaluate neuronal survival and neurological recovery in mice with deficient phagocytosis. The primary outcome of this study will be forelimb function assessed with the staircase test. Secondary outcomes constitute Rotarod performance, stroke volume (quantified on MR imaging or brain sections, respectively), diffusion tensor imaging (DTI) connectome mapping, and histological analyses to measure neuronal and microglial densities, and phagocytic activity. Male mice aged 10-12 weeks will be used for experiments.

## Introduction

Stroke is the second leading cause of death and a major cause of disability worldwide. Following a sudden interruption of the blood supply, there are two major regions of injury within the brain: the ischemic core, where severe hypoperfusion causes rapid cell death and the comparatively less hypoperfused ischemic penumbra, where cells may be stressed and dysfunctional but viable ^1^. Within the ischemic penumbra, the fate of individual neurons results from a complex interplay of numerous biochemical and cellular events, which may lead to irreversible cell death or survival and tissue repair. Among those, activation of microglia, the primary immune effector cells of the brain, or recruited macrophages is a key feature of the pathophysiology of cerebral ischemia. Apart from their potential to release a number of pro- and anti-inflammatory and cytotoxic molecules, activated microglia demonstrate many characteristics of macrophages, including amoeboid-like morphology, capacity to migrate, antigen presentation, and phagocytic activity ^2, 3^. Consequently, one of the main effector functions of microglia is the phagocytic removal of cell debris or dying cells. In these cases phagocytosis is beneficial, because it prevents the disintegration of apoptotic cells and induces an anti-inflammatory response in microglia, thereby contributing to tissue homeostasis and repair (for review see Brown & Neher, 2014) ^4^.

**Figure 1.**
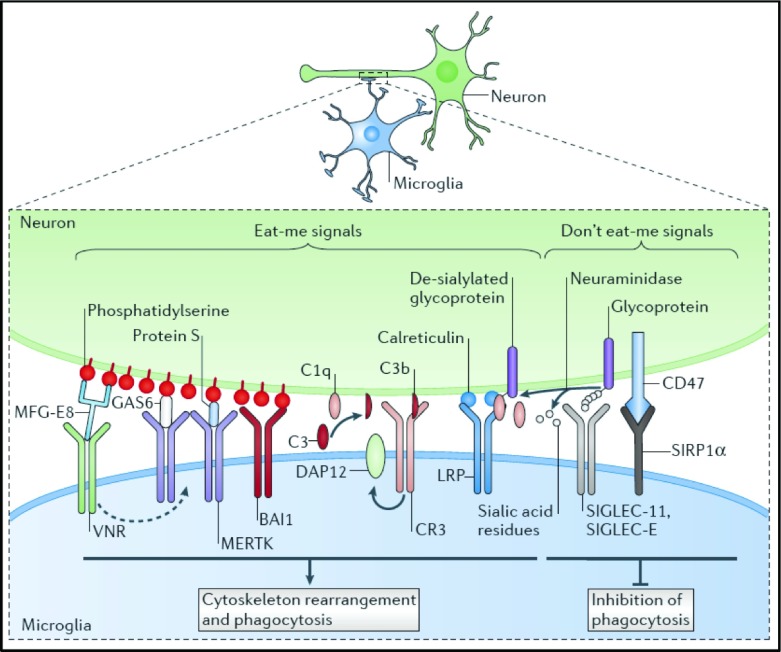
Signalling pathways implicated in the phagocytosis of neurons and neuronal structures. Microglial phagocytosis of neurons is regulated by the neuronal presentation and microglial recognition of ‘eat-me’ (left) and ‘don‘t eat-me’ (right) signals. [Figure and legend reproduced with permission from: Brown GC & Neher JJ. *Microglial phagocytosis of live neurons.* Nat Rev Neurosci 2014 ^4^].

Microglial phagocytosis is closely controlled by the expression of specific cell surface ligands. For the phagocytic removal of host cells, specific ligands, so-called ‘eat-me’ signals, need to be displayed on their surface ^5^ ( Figure 1). Among a wide variety of ligands, exposure of phosphatidylserine (PS) is the best-described and most commonly observed eat-me signal. Importantly, PS externalization of neurons can occur reversibly in response to cellular stress that is not sufficient to induce cell death. In particular, stressed but viable neurons can reversibly externalize PS when exposed to non-toxic levels of oxidative or nitrosative metabolites (as may be released by microglia during inflammation) ^6– 8^, low levels of glutamate ^9^, or growth-factor withdrawal ^10^. In the presence of microglia this PS exposure leads to neuronal engulfment and death, whereas in the absence of microglia, these stressed neurons are able to re-internalise PS and survive ^6, 9^. Neuronal PS can be recognised by different microglial receptors, leading to a cascade of intracellular events that eventually results in the uptake of the ‘eat-me’ signal-exposing neuron ^11^. PS can be recognised directly through microglial transmembrane receptors or indirectly through binding of soluble opsonins, such as milk fat globule EGF-like factor 8 protein (MFG-E8, also known as lactadherin or SED1) ^12^. MFG-E8, a glycoprotein produced by microglia and astrocytes during inflammation, simultaneously engages exposed PS and the microglial vitronectin receptor (the heterodimeric integrin α _ν_β _3/5_), thereby activating phagocytosis ^12, 13^. Similar to MFG-E8, two other opsonins, growth arrest specific gene 6 (Gas6), and Protein S, which also bind to exposed PS on neurons, are in turn recognized by the membrane protein Mer receptor tyrosine kinase (MerTK) ^14^. Of note, MerTK can also be activated downstream of the vitronectin receptor, indicating convergence of the MFG-E8 and MerTK pathways.

**Figure 2.**
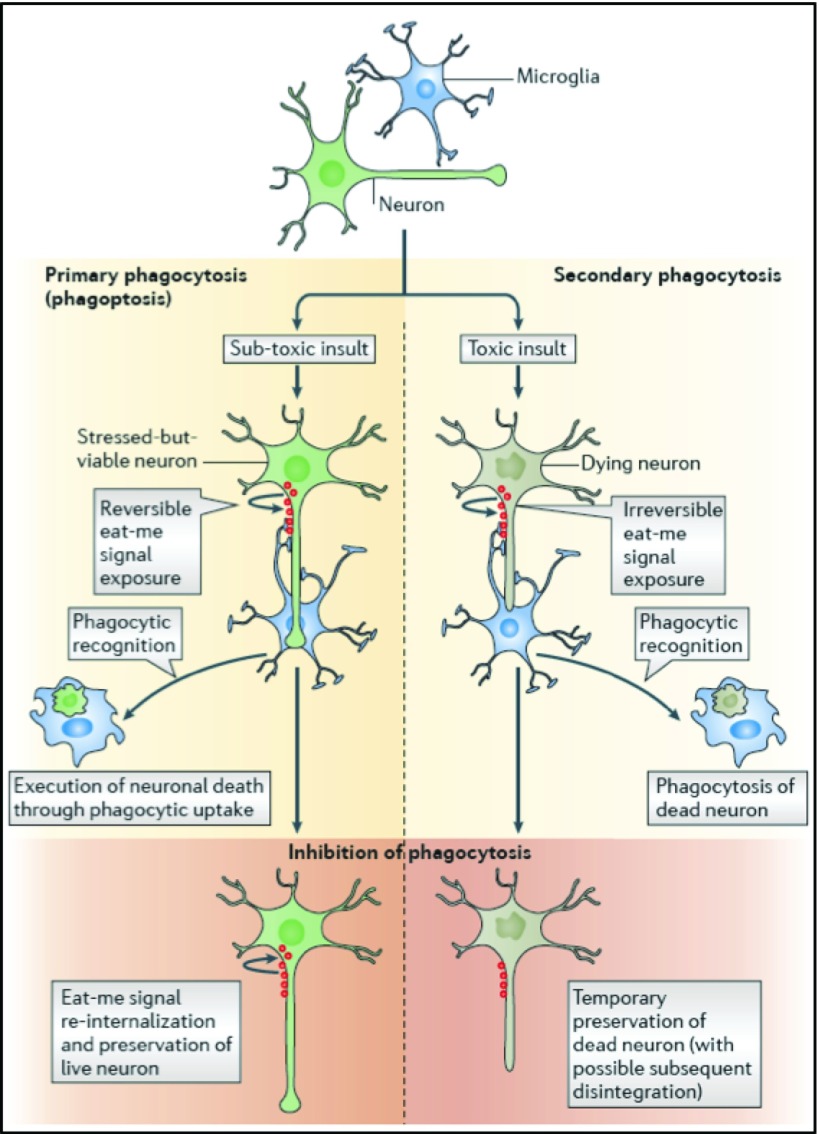
Phagocytosis and phagoptosis. Recent data indicate that phagocytosis can execute the death of viable neurons during development, inflammation, and neuropathology. This form of cell death is called phagoptosis, which means that cell death is caused by the cell being phagocytosed, with the defining characteristic that inhibition of phagocytosis or phagocytic signalling prevents cell death. Experimentally distinguishing between primary phagocytosis (that is, phagoptosis) and secondary phagocytosis (that is, the phagocytosis of a cell dying by apoptosis or necrosis) is possible through inhibiting phagocytosis, which in the first case will leave live cells, whereas in the second case it will leave dead cells (at least temporarily before their disintegration). [Figure and legend reproduced with permission from: Brown GC & Neher JJ.; Nat Rev Neurosci 2014 ^4^].

Traditionally, phagocytosis has been regarded to occur secondary to a target cell being dead or dying. However, accumulating evidence suggests, that during neuroinflammation or cerebral ischemia phagocytes can also eat viable neurons, and thereby induce cell death (for review see Brown & Neher, 2014) ^4^. This form of cell death resulting from the cell being phagocytosed has been termed ‘phagoptosis’ ^15^, with the defining characteristic that inhibition of phagocytosis prevents cell death ( Figure 2). Using a rodent model of focal cerebral ischaemia induced by stereotactic microinjection of the vasoconstrictive peptide endothelin-1 (ET-1) into the striatum or sensorimotor cortex of rats or mice, respectively, we previously found that the phagocytic proteins MFG-E8 and MerTK were transiently upregulated by microglia within the ischaemic area peaking at 3–7 days after insult. Animals deficient for MFG-E8 or the microglial phagocytic receptor MerTK had reduced brain atrophy and improved neurological function. While the number of microglial cells and the levels of inflammatory mediators were indistinguishable between genotypes, microglia from *Mfge8* and *Mertk* knockout animals showed reduced phagocytosis of neurons ^9^. In conclusion, these results suggest that deficiency of MerTK or MFG-E8 blocks phagocytosis of neurons by microglia and thereby prevents engulfment-induced neuronal death. However, the observed behavioural benefits among phagocytosis-deficient animals were moderate at best and the ET-1 ischemia model may have confounding effects on neuroinflammation and neuronal survival as ET-1 receptors are also expressed by neurons, astrocytes, and microglia ^16, 17^.

We therefore propose to investigate how phagocytosis and specific phagocytic signalling pathways contribute to the pathophysiology of stroke, by using an established model of focal cerebral ischemia. We will perform histological, biochemical, and behavioural analyses of phagocytosis-deficient wildtype mice and homozygous *Mfge8* and *Mertk* knockout mice, and use pharmacological inhibition of the MFG-E8 receptor to assess whether phagocytosis is beneficial or detrimental for neuronal survival and neurological function following temporary (45min) middle cerebral artery occlusion (tMCAo). In these animals, we will test:

1)     Whether phagocytic deficiency is beneficial or detrimental for neurological function; and

2)     Whether phagocytic microglia and recruited macrophages contribute to neuronal and/or synaptic loss following cerebral ischemia and if this is beneficial or detrimental for tissue recovery.

By pre-registering this study we strive to foster transparency about our aims, study design, and analysis plan, thereby strengthening the robustness and accountability of our data.

## Methods

### Animals, housing and husbandry

All animal experiments will be performed in accordance with local regulations, and have been approved by the Berlin governmental authorities (Landesamt für Gesundtheit und Soziales, LaGeSo), approval number G057/16.

Male C57BL/6NCrl mice will be derived from Charles River at the age of 8 weeks. Phagocytosis-deficient *Mertk* (Jax: B6;129- *Mertk ^tm1Grl^*/J) and *Mfge8* (from C. Théry, INSERM 932, France) ^18^ knockout mice will be derived from The Jackson Laboratory and Hertie Institute for Clinical Brain Research, respectively, and bred locally. Male homozygous *Mertk* and *Mfge8* knockout mice and their homozygous wildtype littermates will be used in experiments at the age of 10 – 12 weeks. Animals will be group-housed with *ad libitum* access to food and water and cages will be equipped with environmental enrichment tools (red transparent plastic nest box and brown paper towels). Animals will be kept in specific pathogen free (SPF) conditions under a 12 h light/dark cycle (lights on: 8am; lights off: 8pm). Room temperature will be maintained at 22 ± 1°C.

### Methods to prevent bias

Animals will be randomized using the GraphPad calculator tool ( http://www.graphpad.com/quickcalcs/randomize1.cfm) by a researcher who is not involved in the surgical procedure, behavioral, histological, biochemical or MRI analysis. Animals will be allocated to blocks. Blocks will be randomized for a given day of surgery (10 animals) and for a given week of experiments (30 to 40 animals) or 3 – 4 surgery days so that MCAo/sham procedures and drug treatment are randomly allocated to animals and performed in a randomized order. Information on genotype and treatment group allocation will be concealed from experimenters until the end of the study. Behavioral, histological, biochemical and MRI analysis will be performed by researchers who are not involved in the surgical procedure.

### Exclusion criteria

Animals will be excluded from this study if: i) infarct volume is not detected and/or non-middle cerebral artery territory ischemia (such as cerebellar infarction) is detected on MRI imaging at 24h following cerebral ischemia; ii) pellet-reaching performance in the Staircase test differs by more than 1.2 standard deviations from the mean performance of the corresponding genotype and the treatment group at the end of the conditioning phase.

### Anesthesia

All animals will be induced with 2% isoflurane in oxygen and nitrous oxide (ratio 0.3/0.7) and maintained with 1% isoflurane during surgical or imaging procedures.

### Temperature monitoring and body weight measurement

Temperature will be maintained at 37 ± 0,5°C throughout the surgery or imaging procedures using a homeothermic feedback system. In addition, following cerebral ischemia body temperature and weight will be measured once daily. Temperature measurements will be obtained non-invasively using subcutaneous radio-frequency identification (RFID)-transponders (IPTT-300; Bio Medic Data Systems). Transponders will be implanted by subcutaneous injection at least one week prior to the induction of cerebral ischemia.

### Temporary filamentous middle cerebral artery occlusion

Mice will be subjected to 45 minutes filamentous temporary middle cerebral artery occlusion (tMCAo), and will be killed for histological and biochemical analyses after 3, 5, or 21 days, respectively. The filamentous tMCAo model will be performed using Dirnagl *et al*’s method (doi: 10.1038/npre.2010.3492.2) as implemented in our laboratory ^19– 21^. For pain relief, Bupivacaine gel is topically applied in the wound, and the wound is temporally closed with an adaptive suture. Following tMCAo animals will have access to mashed chow placed on the cage floor in a petri dish for up to 24 hours. There will be no additional fluid replacement.

### Inhibition of phagocytic signaling

For inhibition of phagocytic signaling mice will be treated with EMD121974 (Peptide Special Laboratories, GmbH), a commercially available vitronectin receptor-antagonist. EMD121974 or inactive control peptide cRGDfV (Peptide Special Laboratories, GmbH) will be administered daily by intraperitoneal injection for 7 days after tMCAo. The first dose of EMD121974 or control peptide (30 mg/kg) is administered intraperitoneally 6 h after tMCAo, followed by daily doses of 10 mg/kg, respectively.

### Magnetic resonance imaging, stroke volumetry, and connectomics

Magnetic resonance imaging (MRI) will be performed at 24 h and 21 days following tMCAo using a 7 Tesla rodent scanner (BioSpec 70/16AS, Bruker BioSpin, Ettlingen, Germany) with a 16 cm horizontal bore magnet and a 9 cm (inner diameter) shielded gradient with an H-resonance frequency of 300 MHz and a maximum gradient strength of 300 mT/m. For imaging, a quadrature volume resonator with an inner diameter of 86 mm for excitation and a decoupled mouse head quadrature surface coil for signal reception (both Bruker) will be used. Data acquisition and image processing will be performed with the Bruker software Paravision 6.0.1. and custom MATLAB (MathWorks, Natick, MA, USA) scripts. Both, at 24 h and 21 d, a rapid acquisition with relaxation enhancement (RARE) T2-weighted (T2w) sequence will be used for anatomical imaging (repetition time TR=3500, echo time TE=33 ms, RARE factor 8, 4 averages, 32 contiguous coronal slices, slice thickness 0.5 mm, field of view FOV=1.92 × 1.92 cm ^2^, matrix MTX=192×192, total acquisition time TA=5:36 min). At 21 d, in addition to anatomical imaging, diffusion tensor imaging (DTI) using a Stejskal-Tanner 4-shot spin echo EPI pulse sequence will be performed (TR/TE=3500 ms/28.5 ms, 1 average, geometry identical to the T2w image, MTX=96×96, high angular resolution diffusion (HARDI) encoding scheme with 60 diffusion directions, b=1000 s/mm ^2^, five b=0 images, diffusion duration δ=2.6 ms, diffusion separation Δ=8.5 ms, TA=15:10 min). Maps of fractional anisotropy and mean/axial/radial diffusivity (FA, MD, AD, RD) will be calculated and the DTI connectome of each mouse will be reconstructed using whole brain fiber tracking in DSI studio ( http://dsi-studio.labsolver.org/) as described previously ^22^. The stroke lesion will be segmented on T2w images 24 h post-surgery using Analyze 10.0 software (AnalyzeDirect, Overland Park, KS, USA) by selecting hyperintense areas of ischemic tissue by an experienced researcher and a mask will be exported. The 24 h lesion mask, and 21 d DTI parameter maps will coregistered to the Allen mouse brain atlas using their corresponding anatomical T2w images in the MATLAB toolbox ANTX ( https://github.com/philippboehmsturm/ANTX/) ^23– 25^. Since the registration includes nonlinear terms, lesion volumes are effectively corrected for edema when measured in atlas space. Edema-corrected lesion volume and DTI parameters will be measured in all regions of the atlas and in a volume of interest encompassing the lesion territory at 24 h. Values will be compared using two sample t-test using false discovery rate post-hoc correction. ANTX will also be used to coregister a modified atlas with less brain structures (MRMNeAt) to the DTI connectome and a graph theoretical analysis will be performed using the brain connectivity toolbox followed by comparison of scalar network parameters as described previously ^22, 26^.

### Modified DeSimoni neuroscore

DeSimoni’s neuroscore ^27^, a composite of general behavioral alterations and focal motor, sensory, reflex, and balance deficits, will be performed at days 1, 2, 7, 14 and 21 following cerebral ischemia as implemented in our laboratory ^19^. In brief, general health and behavioral alterations and specific focal deficits will be scored separately and subsequently added to form a summation score. The maximum score is 43 points, with more points meaning more deficits.

### Rotarod

The Rotarod, a widely used test of motor coordination and learning, will be performed at days 2, 7, and 14 following cerebral ischemia as implemented in our laboratory ^19^. The best run of three runs per time point will be used for statistical analysis.

### Staircase

The Staircase test will be performed daily for 7 days before and up to 21 days after cerebral ischemia for conditioning and testing skilled forelimb motor function, respectively. Mice will be placed in a Plexiglas holding box with an attached baited double staircase. Food rewards are presented bilaterally on a descending double staircase with each of the 8 steps containing a food-well loaded with 20 mg sucrose pellets (Sandown Scientific). During testing, the animal is required to ascend onto a central base with one staircase on either side, designed such that only the ipsilateral paw can reach a given staircase. A lip on the platform edge prohibits the animal from dragging the pellet up alongside the platform, thus requiring it to be grasped and brought up and around the platform edge. The pellets in lower wells are more difficult to grasp than those in wells higher on the staircase, thus providing objective measures of maximum forelimb extension and grasping skill. Outcome is measured as the number of pellets grasped and eaten with each forepaw. Animals are tested once daily for 20 minutes per session.

### Quantification of infarct volume, cellular densities, and phagocytosis

Following perfusion with physiological saline and 4% paraformaldehyde (PFA) and cryosectioning of the fixed tissue, brain sections will be stained for cresylviolet or NeuN to label all surviving cells and infarct size will be determined by stereological quantification by a blinded observer on random sets of every 12 ^th^ systematically sampled 40 μm thick sections throughout the brain. Analysis will be conducted using the Stereologer software (Stereo Investigator 6; MBF Bioscience) and a motorized x-y-z stage coupled to a video microscopy system (Optronics) as previously described ^28^, with application of the Cavalieri estimator technique ^29^. Neuronal and microglial/macrophagic densities will be quantified using the optical fraction fractionator on sections stained for NeuN and ionized calcium binding adaptor molecule 1 (Iba1), respectively ^30^. To quantify phagocytosis of neurons, high-resolution confocal z-stack images of the peri-infarct area stained for microglia and recruited macrophages (Iba1) and neuronal nuclear antigen (NeuN) will be obtained. Z-stack acquisition will be followed by 3-dimensional reconstruction using Imaris software (Bitplane, UK) and quantifying the percentage of microglia/macrophages that contain NeuN-positive inclusions (as described in detail in 9).

### Sample size calculation and statistical analysis

The study is designed with 80% power to detect a relative 25% difference in pellet-reaching performance in the Staircase test. *A priori* power analysis using a repeated measures ANOVA with Tukey’s post hoc test under the following assumptions α = 0.05, β = 0.2, mean, SD 20% of the mean determines the number of required experimental units at 17 animals per group. Based on previous results with this model we estimate that 15% of animals will have to be excluded from this study based on insufficient infarct volume (10%) or insufficient pellet-reaching performance in the Staircase test, respectively. Thus, we will use 20 animals per genotype or treatment group, respectively ( *Mertk* and *Mfge8* phagocytosis-deficient knockout animals vs. littermate controls and phagocytosis inhibitor-treated animals vs. untreated controls, respectively). Data will be tested for normal distribution using D’Agostino’s *K* ^2^ test and analyzed by 1-way analysis of variance (ANOVA) or in case of measuring the effects of 2 factors 2-way ANOVA with posthoc Holm-Sidak adjustment for p values. For DTI parameter maps, lesion volumes, and network analysis metrics a false discovery rate to control for multiple comparisons and Type I errors will be used. If only 2 groups are compared, unpaired, two-tailed Student’s t test will be used. Nonparametric functional data will be compared using Kruskal-Wallis test with posthoc Dunn multiple comparison test. Survival will be compared for the effect of genotype or treatment using a log-rank Mantel–Cox test. P values ≤ 0.05 are considered statistically significant.

### Study design

Following tMCAo, animals will be killed for histological and biochemical analyses after 3, 5, or 21 days, respectively. Functional, MRI, and histological outcome data of phagocytosis-deficient *Mertk* and *Mfge8* knockout animals and littermate controls will be assessed using a block design (i.e. all animals will be tested before data analysis is performed). Outcome data of phagocytosis inhibitor-treated animals vs. untreated controls will be assessed using a group sequential design with 4 stages (k=4, sample size at stage 1, 2, and 3, respectively: 14 animals (7 animals vs. 7 animals), sample size at stage 4: 12 animals (6 animals vs. 6 animals), for details see 31). To save animals and money, testing will be terminated following unblinded interim data analysis at each stage based on the following stopping criteria: stage 1) none or detrimental effect of phagocytosis inhibitor on neuronal and/or synaptic survival and tissue recovery at day 5 following tMCAo; stages 2–4) detrimental or beneficial effect of phagocytosis inhibitor on pellet-reaching performance in the Staircase test following tMCAo (i.e. mean pellet-reaching performance differs by more than 20% from one standard deviation of the mean pellet-reaching performance of the control group).

### Study timeline

The study will be conducted within 12 months, following successful peer review of this Stage 1 Registered Report submission.
