# Supplementary material for: Stage 1 Registered Report: Effect of deficient phagocytosis on neuronal survival and neurological outcome after temporary middle cerebral artery occlusion (tMCAo)
Source: F1000Res. 2018 May 16;6:1827. Originally published 2017 Oct 12. [Version 3] doi: 10.12688/f1000research.12537.3 (PMC5664978; doi:10.12688/f1000research.12537.3)
Supplement: Peer review history

# Peer review: Referee response for version 1

**DOI:** 10.5256/f1000research.13575.r26912

**Published:** 2017-10-31

**Authors:** Ádám Dénes

**Affiliations:** 1 MTA Institute of Experimental Medicine, Hungarian Academy of Sciences, Budapest, Hungary

I find it a great idea to share the design of the proposed studies with the scientific community. In general, the aim of the research is great and the methods to be used are largely appropriate. The authors deserve the credit for providing details on given experimental objectives, including randomisation, blinding, power calculation and desrciption of the experimental procedures, which all increase the transparency and credibility of these studies.

 Specific points:

 1. It is stated that the studies will be completed in 12 months, which is doable given that all mouse strains are available and breeding on site. Based on what is described in the methods, it remains unclear how long it will take to establish breeding colonies from the phagocytosis-deficient strains derived from The Jackson Laboratory and how the phenotype of these will be compared to the control animals, which will be obtained from another vendor (Charles River). For such studies comparing different KO lines, it would be useful to ensure the identitiy of the background ideally by establishing heterozygous breeding colonies and use littermates for the experiments. C57BL/6 (and other) mouse stains from different vendors could show markedly different inflammatory or phagocytic responses that could influence the interpretation of the results.

 2. In addition to using Mfge8 KO mice, the pharmacological inhibition of the microglial MFG-E8 receptor is also proposed in the methods. If phagocytic signaling will be blocked by intraperitoneal administration of the antagonist, will this regimen effectively reach areas of the brain where there is no major BBB injury? Details on brain penetration and pharmacokinetics of the drug (if known) would be useful.

 3. The phagocytosis-deficient lines and pharmacological inhibitors will likely change phagocytic responses in all tissue macrophages, including perivascular macrophages in the brain (the latter will possibly be influenced by the pharmacological treatment earlier than microglia).  Unless the authors plan sophisticated studies on descriminating microglial actions from that of blood-borne phagocytes that may be recruited into the affected brain areas, it may be mose useful to talk about microglia /macrophages throughout.  Iba1 is not an ideal marker either to discriminate brain microglia /macrophages of different origin.

 4. Several excellent sensory-motor tests are planned for functional assessment, among which the staircase requires extensive conditioning and testing. Has the same battery of tests been successfully used in similar MCAo studies previously? Extensive testing might lead to overtraining of mice and a reduction of differences in performance between experimental groups in spite of the expected changes in the number of surviving neurons.

 The notes above merely aim to help the interpretation of these excellent studies rather than suggesting extensive changes in experimental design, particularly regarding  costly breeding and determination of microglial-specific effects.

**Have the authors pre-specified sufficient outcome-neutral tests for ensuring that the results obtained can test the stated hypotheses, including positive controls and quality checks?**

 Yes.

I have read this submission. I believe that I have an appropriate level of expertise to confirm that it is of an acceptable scientific standard.

# Response

**Published:** 2017-11-09

**Authors:** Julius Emmrich

**Affiliations:** Charité-Universitätsmedizin Berlin, Germany

We would like to thank the reviewer for appreciating the aim of this registered report and the helpful comments. Where appropriate the manuscript has been revised following the reviewer’s remarks.

 Point 1: We agree with the reviewer that the description of breeding procedures lacked some detail. Heterozygous breeding colonies for phagocytosis-deficient mouse strains have already been established in our laboratory. Homozygous Mertk and Mfge8 knockout offspring will be used in experiments. Homozygous wildtype littermates will serve as controls. C57BL/6NCrl mice, which will be obtained from Charles River, will be used for inhibition experiments.

 Point 2: Several pharmacokinetic studies have shown penetration of EMD121947 (cilengitide) through the intact blood brain barrier. An excellent study on the pharmacokinetics of cilengitide in mice and men by Dolgos et al. can be found here: www.ncbi.nlm.nih.gov/pmc/articles/PMC4804314.

 Point 3: We thank the reviewer for bringing up this important point. We do not plan to discern effects of deficient phagocytosis between microglia and blood-derived macrophages. As suggested by the reviewer, we have modified the wording throughout the manuscript.

 Point 4: We appreciate the reviewer’s concern that excessive training might have a negative impact on behavioral outcomes. Both tests have been established in our laboratory and have been used in parallel for MCAo studies. Several other groups have also used a combination of these tests following MCAo in mice (see for example: http://www.jneurosci.org/content/24/27/6209). It is well conceivable that training in one test might have an influence on the results of the other. The staircase and Rotarod tests assess skilled forelimb function (i.e. number of pellets grasped and eaten with each forepaw) and general motor coordination, respectively. Thus, we believe that the additional information which is gained by assessing two distinctly different motor skills outweighs a potential confounding effect.  

# Peer review: Referee response for version 1

**DOI:** 10.5256/f1000research.13575.r26908

**Published:** 2017-10-18

**Authors:** Stuart M. Allan

**Affiliations:** 1 Faculty of Biology, Medicine and Health, The University of Manchester, Manchester, UK

The rationale behind the study is well explained and on the most part the methods are described in full. Importantly the authors provide full details of experimental design, including power calculations and planned statistical analyses. They also propose the use of sequential design which is very unusual in preclinical research, but can be used more widely.

 Overall there are no major concerns regarding the planned study but the report would benefit from additional detail and clarification in a few places as detailed below:

1. The authors do not specify what will be their primary endpoint and what will constitute secondary analyses. The study is powered on the Staircase test which would imply that this is being taken as the primary outcome. The title and abstract should be amended to clarify this, and the latter should also have details of the secondary outcomes that are being assessed.
2. The abstract would benefit from additional details on the age and sex of mice used.
3. Page 2: The authors indicate no grants were involved in supporting the work. It is possible to clarify therefore exactly how the work was supported, since presumably the funds had to come from somewhere.
4. Page 5: Clarify if a homeothermic feedback system used to maintain body temperature.
5. Page 5: Clarify if modified diet provided to aid post-stroke recovery and on similar lines if fluid replacement instigated.
6. Page 5: Clarify why systemic analgesia not used.
7. Page 5: Clarify how dosing regimen decided on.
8. Page 5: Clarify why only male mice being used.
9. Page 6: For rotarod clarify why best run being used rather than average.
10. Page 6: Please specify endpoints (days post MCAo) when animals are perfused.
11. Page 7: day 5 has not been previously specified as an endpoint.

**Have the authors pre-specified sufficient outcome-neutral tests for ensuring that the results obtained can test the stated hypotheses, including positive controls and quality checks?**

 Yes

I have read this submission. I believe that I have an appropriate level of expertise to confirm that it is of an acceptable scientific standard.

# Response

**Published:** 2017-11-09

**Authors:** Julius Emmrich

**Affiliations:** Charité-Universitätsmedizin Berlin, Germany

We would like to thank the reviewer for the insightful and constructive remarks. All points have also been addressed in the revised version of this article.

Point 1: The primary outcome measure of this study is functional recovery following tMCAo as determined with the staircase test. The study has been powered accordingly. Secondary outcomes constitute Rotarod performance, stroke volume (quantified on MR imaging or brain sections, respectively), diffusion tensor imaging (DTI) connectome mapping, and histological analyses to measure neuronal and microglial densities, and phagocytic activity.

Point 2: Male mice at the age of 10-12 weeks will be used for experiments. The abstract has been modified accordingly.

Point 3: Funds for transgenic animal breeding will be provided by the German Federal Institute for Risk Assessment and parts of the study will be supported by grants of the Federal Ministry of Education and Research (BMBF) for the Center for Stroke Research Berlin to UD, CH and PBS and the Berlin Institute of Health, QUEST Center to UD.

Point 4: Temperature will be maintained at 37 ± 0,5 °C during surgery or imaging procedures using a closed loop homeothermic feedback system which consist of a heating blanket connected to a rectal probe.

Point 5: Following tMCAo animals will have access to mashed chow placed on the cage floor in a petri dish for up to 24 hours after surgery. There will be no additional fluid replacement.

Point 6: The brain itself is not sensitive to pain. Local delivery of gel comprising anesthetics is sufficient to reduce the pain that originates from the wound and the vessels. Many inflammatory signals are modulated by non-steroid analgesic drugs or opioids. Therefore, we aimed to avoid using drugs that probably affect stroke outcome or inflammatory signaling including phagoptosis.

Point 7: The dosing regimen of EMD121947 (cilengitide), an inhibitor of phagocytic signaling, was decided based on results from previous pharmacokinetic studies in mice and preliminary, unpublished data.

Point 8: Only male mice will be included in this study, which might introduce a potential sex bias. However, including female animals is likely to increase variation, which, in turn, would require the use of additional animals, time, and effort. The number of subjects for this study was limited to the minimum required to obtain scientifically meaningful results and only the use of male mice has been approved by the Berlin Ethical Review Panel (LaGeSo). If results from the pharmacological intervention study will be encouraging, we would consider planning a pre-clinical trial that is powered to study sex differences by EMD121947.

Point 9: We expect that the variation of Rotarod results will be smaller if the best run out of three trials is used rather than the average. If we would calculate averages across three trials, an accidental slip or drop off the rod that is caused by lack of attention or motivation would falsely increase variation. We believe that this approach is best suited to obtain adequate information on the motor coordination of a given animal.

Point 10: As specified on page 5 in the Materials and Methods section, endpoints will be at 3, 5, or 28 days following 45min tMCAo, respectively.

Point 11: As specified on page 5 in the Materials and Methods section, animals will be killed for histological and biochemical analyses after 3, 5, or 28 days after 45min tMCAo, respectively. To improve clarity, this information has now also been included on page 7 (study design).
